# The rate of venous thromboembolism after knee bone marrow concentrate procedures: should we anticoagulate?

**DOI:** 10.1007/s00264-022-05500-3

**Published:** 2022-07-18

**Authors:** Christopher J. Centeno, Brandon T. Money, Ehren Dodson, Ian Stemper, Neven J. Steinmetz

**Affiliations:** 1 Centeno-Schultz Clinic, Broomfield, CO USA; 2 Regenexx, LLC, Research and Development, Broomfield, CO USA

**Keywords:** Knee osteoarthritis (OA), Bone marrow concentrate (BMC), Venous thromboembolism (VTE), Total knee arthroplasty (TKA), Deep vein thrombosis (DVT), Pulmonary embolism (PE)

## Abstract

**Purpose:**

Intra-articular injections of autologous, minimally manipulated, cell therapies such as bone marrow concentrate (BMC) to treat knee osteoarthritis (OA) may delay or prevent future total knee arthroplasty (TKA). Arthroplasty has the known and substantial risk of venous thromboembolism (VTE) and requires routine prophylaxis, whereas the VTE risk associated with knee BMC injections is unknown. We report on the rate of VTE from a large orthobiologics patient registry and assess whether knee BMC procedures require routine prophylaxis.

**Methods:**

A retrospective analysis of knee osteoarthritis cases tracked in a treatment registry and treated at 72 clinical sites with BMC from 2007 to 2020 who were not prophylactically anticoagulated was performed to identify adverse events (AEs) associated with VTE. Treating physicians were contacted to improve discovery of possible occurrences of VTE.

**Results:**

Twenty cases (0.16%) of VTE were identified from the registry of 12,780 knee BMC treatments. These events were less frequent than the published data demonstrate for anticoagulated TKA patients.

**Conclusion:**

Based on the rates of VTE from our retrospective treatment registry analysis compared to the risk of medication-induced haemorrhage, routine prophylactic anticoagulation is not recommended for intra-articular knee BMC procedures. Further research into safety and efficacy of BMC treatment for knee OA is warranted.

**Clinical trial identifier:**

NCT03011398, retrospectively registered.

## Introduction

The utilization of autologous cell-based therapies, including bone marrow concentrate (BMC), continues to be studied for treatment of orthopaedic conditions including knee osteoarthritis (OA). BMC is comprised of a heterogenous mix of nucleated cells which include mesenchymal stem cells, in addition to platelets, growth factors, cytokines, and extracellular vesicles which may play a role in the facilitation of tissue healing [1]. Early research has shown encouraging results that BMC treatment may enable prolonged function and reduced pain in knee OA patients, potentially delaying or preventing the need for surgical intervention [2–4].

Low rates of serious adverse events (SAEs) in patients receiving BMC injections for knee OA have been reported in a large case series and systematic review [5, 6]. It has also been reported in two large case series that no increased risk of tumour formation exists in patients who underwent BMC treatment for orthopaedic conditions [6, 7]. However, a detailed analysis of the rate of venous thromboembolism (VTE) after knee BMC treatment has not yet been explored.

Total knee arthroplasty (TKA) for symptomatic end-stage knee OA is associated with significant morbidity [8]. The annual incidence of these surgical procedures in the USA is projected to increase over the next few decades [9–12]. However, a recent large systematic review found a SAE rate of 4.8% and a 30-day hospital readmission rate of 7.2% following primary TKA [13]. In addition, VTE, comprised of deep vein thrombosis (DVT) and pulmonary embolism (PE), has been identified in 40–84% of post knee surgery courses, including TKA, when aggressive prophylaxis is not implemented [14–16]. Prophylactic anticoagulation with medications including aspirin, low molecular weight heparin, warfarin, and newer direct oral anticoagulants (DOACs) following TKA have resulted in significant reductions of reported VTE incidence [17–19]. However, the risk of bleeding from anticoagulation is a concern. Gastrointestinal (GI) and surgical site bleeding are among the most commonly seen events while rare cases of intracranial haemorrhage (ICH) are possible [20].

In the present investigation, we report the frequency of VTE events following BMC injections of the knee utilizing 13 years of registry data collection and compare that to TKA rates. We also discuss the risk of bleeding associated with anticoagulation to determine if the potential benefits of VTE prophylaxis outweigh the risks.

## Methods

All patients receiving orthobiologic treatments who consented were tracked in a formal registry (OHRP #IRB00002637) comprised of 72 US-based clinics. Upon enrollment into the registry, patients were prospectively tracked using an electronic data capturing system (ClinCapture software, Clinovo Clinical D Solutions, Sunnyvale, CA; then later Dacima Software, Montreal, Quebec). As of the time of publication, over 33,000 patients have enrolled into the registry, of which a subset of patients are knee OA patients treated with BMC. The registry collects pre- and post-procedure pain and functional levels from self-reported joint-specific questionnaires via emailed surveys including details about whether the patient underwent subsequent surgery or experienced any AEs. Follow-up survey time points include one, three six, 12, 18, and 24 months, followed by annual surveys up to 20 years. Cases comprised of patients who had their knee(s) treated were included in the study analysis.

To gather comprehensive details about AEs, we used two reporting mechanisms. First, patients were asked, “Did you experience any side effects or complications you believe may be due to the procedure (e.g. infection, illness, etc.)?” If the patient answered affirmatively, they were prompted with questions about the area of the body experiencing the AE (e.g., a particular joint or a systemic response), the type of complication (e.g., pain, swelling, infection, DVT), the intensity (scaled 1–5), onset (ranging from < 1 day to > 14 days after the procedure), and whether it was a pre-existing condition. Patients also provided additional details in a free-form text field. Furthermore, subsequent questions asked if the complications were reported to the treating physician, whether the complications were life-threatening, required hospitalization, and/or required another form of emergency medical attention, and if the event resulted in significant prolonged disability. Reported AEs triggered automated emails to prompt timely review by the treating physician, who assessed whether the event was related to the procedure and/or injectates used.

SAEs are defined as any AE that results in death, is life threatening, results in inpatient hospitalization or prolongation of existing hospitalization, results in a persistent or significant disability/incapacity, or may require intervention to prevent one of the other outcomes listed above [21]. SAEs of interest in this analysis include blood clotting events such as DVT and PE. A legacy registry format used free-form text fields to collect SAE information; therefore, string searches were performed on these fields to identify applicable SAEs. To do this, the text was processed, including conversion to lowercase and removal of punctuation to standardize reporting. The search terms “dvt,” “embolism,” “thrombosis,” and “clot” were used. The current registry format also allows patients to choose from a drop-down menu of a variety of potential AEs, including DVT and PE.

The second reporting mechanism utilized was direct follow-up with physicians providing BMC treatments at clinic-based sites. Since symptomatic VTE events are rare and often necessitate action to protect the patient’s life, we theorized that if an event of this nature were to occur, it would typically be memorable to the treating physician. Hence, all physicians who participated in this registry were surveyed for details on any VTE events recalled during the studied time period. This allowed us to identify additional cases beyond the patient-reported cases in the registry. Physicians were contacted via email up to three times. If there was no response, attempts were made to contact the physician via phone two times, one week apart. Finally, the lead author reviewed all reported VTE events to determine causation using our already published criteria [6]. The inclusion criteria included:

- A confirmed VTE event requiring treatment
- VTE event within 1 month of the BMC procedure
- Relatedness to the procedure

## Results

A total of 12,780 knee BMC injection cases were identified in the registry between 2007 and 2020. Table 1 provides patient demographic information regarding these cases. Using the previously outlined registry search criteria, we found 39 potential events. A total of 126 physicians were contacted for VTE reporting. Eight physicians reported a VTE event and five cases overlapped between the registry and the physician reporting lists, leaving three additional patients included in the reporting.

**Table 1:** Patient demographics of registry patients with knee BMC treatment

Variable	*N*	% or mean ± SD
Gender	12,780	
Male	6037	47%
Female	5059	40%
Unknown	1684	13%
Age	11,115	59 ± 13
BMI	10,643	28.0 ± 5.7

**Fig. 1:**
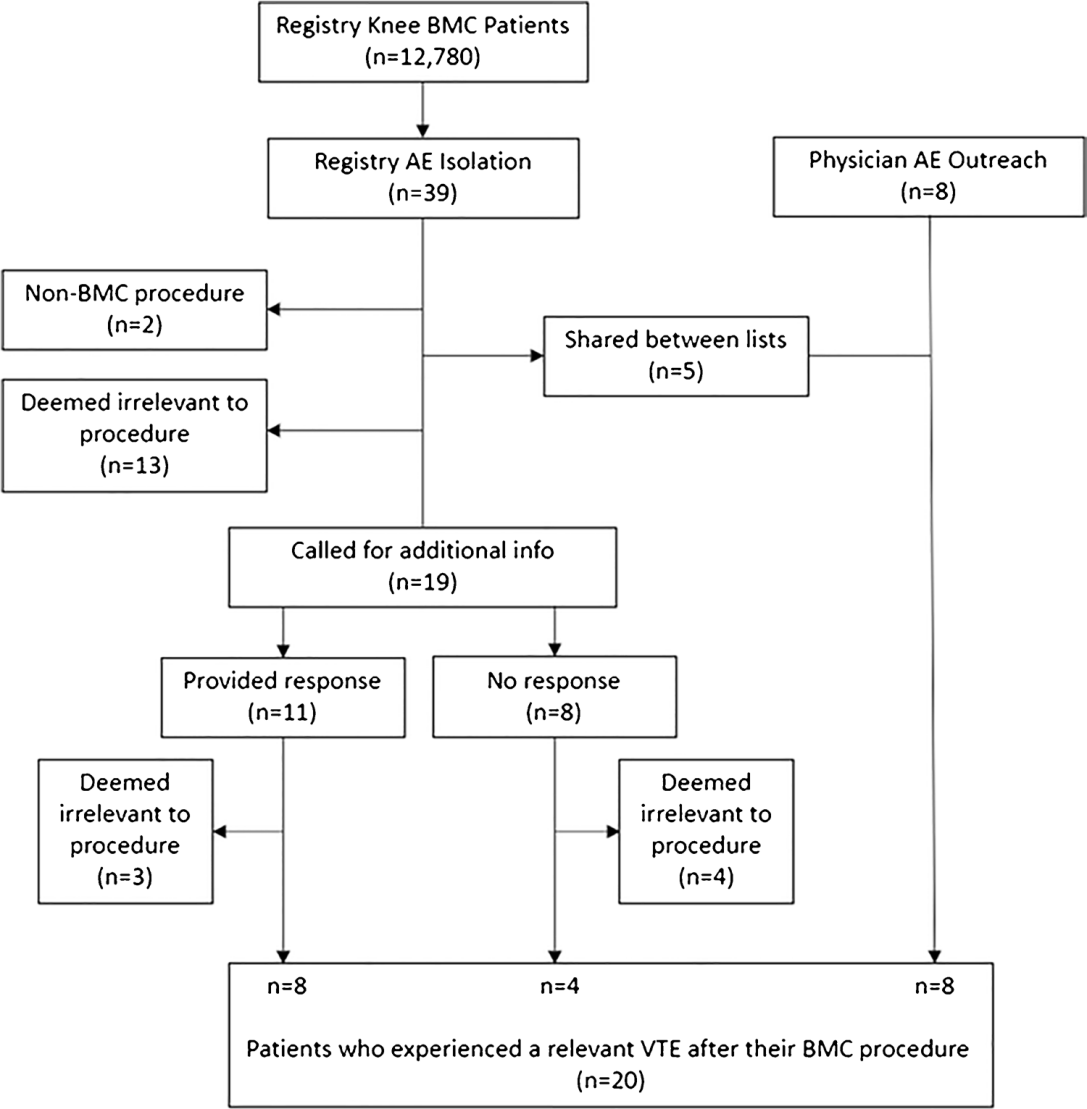
Flow chart describing the process in which VTE events were identified as relevant to BMC procedure following physician adjudication by the lead author. Patients who were called for more information, but did not respond, were still included based on the information collected via the patient registry

From the total of 42 cases, 19 patients met the inclusion criteria and these were contacted for additional information, with 11/19 (58%) responding to contact attempts via phone or email. After re-reviewing medical records and patient responses for causation for all 42 cases, a total of 12 cases of VTE were identified from this group. Eight patients were identified via the physician outreach method, all of which were determined to have causally related VTE. Table 2 shows demographics of the 20 VTE patients determined to be causally related to the knee BMC procedure. Fifteen different physicians were involved in these cases. Figure 1 describes the process in which the 20 cases were identified.

**Table 2:** Patient demographics of reported VTE events

Variable	*N*	% or mean ± SD
Gender	20	
Male	13	65%
Female	7	35%
Unknown	0	0%
Age	20	55.8 ± 13.1
BMI	20	26.9 ± 3.0

The incidence of VTE in this patient population was determined to be 1 in 639 (0.16%). There was one fatal PE in an at-risk patient, resulting in a fatality rate of 1 in 12,780 (0.01%). Additionally, from the available data, 15/20 (75%) patients did not have history of clotting, and 10/20 (50%) were at risk of clotting using already published risk factors [22]. Ten out of the 20 VTE cases (50%) resulted in a PE.

## Discussion

The number of primary TKAs performed has seen a continued upward trend over the last several decades. The incidence rate of knee arthroplasty in the USA is 235/100,000 total population [23]. The growth of these procedures is projected by different models to range from 1.3 to 3.5 million in the USA by 2030 and 1.5 to 6 million by 2050, with most of this cost borne by government-funded healthcare programs like Medicare and Medicaid [9–12]. At approximately $15,000–$20,000 per procedure (exclusive of complications), the cost of 3.5 million TKAs will be roughly $61 billion [24]. Over 40,000 periprosthetic joint infections following TKA are projected annually by 2030 and these alone will cost the US healthcare system over $1 billion [25]. These complication figures warrant the consideration of other safe and efficacious methods in the treatment of symptomatic knee OA such as BMC treatments.

Published findings on BMC injected intra-articularly (IA) or intra-osseously (IO) include several randomized trials and large safety studies. In one study, favourable outcomes in knee OA patients treated with IA BMC were reported when compared against physical therapy [2]. In two IO studies, favourable 15-year results were published when comparing IO to IA and IO to TKA in the same patients [3, 26]. Regarding safety of BMC procedures, a registry analysis by Centeno et al. of over 2300 patients reported an overall rate of SAEs *possibly* related to the procedure of 0.55% with 0.17% deemed definitely related to the procedure [6]. In another study by Hernigou et al. of 1873 patients treated with BMC for various orthopaedic conditions, there was no increased risk of tumour formation or cancer in patients compared to the general population after 12.5 years, on average, post-treatment [7]. While the limited studies that have been published to date suggest that BMC injections may be a treatment that could replace TKA in select patients or possibly extend the time until that surgery is needed in others, with a lower rate of serious complications, additional studies are needed to further confirm this. The important finding in the present study is that we introduce rate data for an additional safety consideration for BMC treatments, the potential for a VTE event in unanticoagulated patients receiving BMC treatments.

Our calculated rates of VTE attributed to the BMC procedure in unanticoagulated patients are low (0.16%), with one death from PE over the course of 13 years of registry data. In the case of the fatal event, the DVT occurred in the contralateral lower extremity and the patient had a personal history of cancer and a family history of DVT. The rates of VTE following treatment of knee OA with BMC in our registry analysis are less than those reported in the literature following TKA even when prophylactic anticoagulation is used. For example, multiple large studies involving patients who underwent TKA with subsequent prophylaxis reported a VTE rate of 1.03–1.42% [17–19]. The symptomatic PE rate in a meta-analysis of over 27,000 post-TKA patients receiving prophylactic anticoagulation was 0.37% [27]. The overall mortality rate following TKA has been reported in two large studies at 0.3% [28, 29].

To determine if routine prophylaxis of VTE for BMC patients is a net positive or not, the risk of VTE in these procedures in unanticoagulated patients would need to exceed the risk of anticoagulation. There are numerous risks associated with medication-based VTE prophylaxis. Major bleeding events were identified in 2.94% of over 30,000 patients who received either aspirin, fondaparinux, enoxaparin, or warfarin following TKA [17]. In a meta-analysis of 43 randomized trials and over 166,000 patients receiving anticoagulation, Miller et al. compared DOACs to other anticoagulants and found the rate of haemorrhage to be similar [30]. A more serious complication is ICH, which carries a mortality rate of approximately 60% and has been reported to occur in 0.06–1.4% of cases based on an analysis of 18 randomized-controlled trials of various anticoagulants [31]. Given that the calculated VTE rate is an order of magnitude less than the rate of bleeding events after anticoagulation, at the present time, routine prophylactic anticoagulation following BMC treatment for knee OA is not recommended.

### Limitations

This study has limitations in patient registry enrollment, registry questionnaire response rates, and the realities of relying on physicians and patients to report AEs. Regarding patient reporting, the overall response rate for our registry is ~ 50% at one month and ~ 60% at three months. Therefore, the possibility exists that registry-enrolled patients may not have reported the AE to their treating physician nor documented it in the registry, causing missing data. Multiple attempts were made at contacting patients diagnosed with post-procedure VTE directly to obtain more information regarding their specific cases. Some were unable to be reached; thus, additional information that could have excluded a potential report was not 100% complete and the report was included to err on the side of caution.

Additionally, physicians participating in the registry may be hesitant to divulge details regarding known VTE events. Repeated efforts were made to contact these physicians, but this was not always successful, leading to possible missing data. While the final estimated total procedure count attempted to account for this issue, there may be discrepancies between total procedures actually performed and those entered into the registry. Meaning that the registry only accepts patients willing to be consented to be tracked. Hence, non-consented patients represent an additional possible source of missing data.

## Conclusion

The frequency of VTE events encountered following knee osteoarthritis treatment with BMC from our registry data analysis is much lower than the frequency of VTE events associated with TKA, even when chemoprophylaxis is added to the surgery. We conclude that knee BMC treatment does not warrant routine anticoagulation, as the risk of major bleeding events exceeds that of VTE.

## Data Availability

Data may be made available upon request.
